# Importance of the plasma soluble HLA-G levels for prognostic stratification with traditional prognosticators in colorectal cancer

**DOI:** 10.18632/oncotarget.16457

**Published:** 2017-03-22

**Authors:** Jing-Bo Li, Yan-Yun Ruan, Bin Hu, Shan-Shan Dong, Tie-Nan Bi, Aifen Lin, Wei-Hua Yan

**Affiliations:** ^1^ Medical Research Center, Taizhou Hospital of Zhejiang Province, Wenzhou Medical University, Linhai, Zhejiang, People's Republic of China; ^2^ Human Tissue Bank, Taizhou Hospital of Zhejiang Province, Wenzhou Medical University, Linhai, Zhejiang, People's Republic of China; ^3^ Department of Gastrointestinal Surgery, Taizhou Hospital of Zhejiang Province, Wenzhou Medical University, Linhai, Zhejiang, People's Republic of China; ^4^ Department of Laboratory Medicine, Xianju People's Hospital, Xianju, Zhejiang, People's Republic of China

**Keywords:** soluble HLA-G, colorectal cancer, prognosis

## Abstract

An increased peripheral soluble HLA-G (sHLA-G) expression has been observed in various malignancies while its prognostic significance was rather limited. In this study, the prognostic value of plasma sHLA-G in 178 colorectal cancer (CRC) patients was investigated. sHLA-G levels were analyzed by specific enzyme-linked immunosorbent assay. Data showed sHLA-G levels were significantly increased in CRC patients compared with normal controls (36.8 U/ml vs 25.4 U/ml, *p* = 0.009). sHLA-G in the died were obviously higher than that of alive CRC patients (46.8 U/ml vs 27.4 U/ml, *p* = 0.012). Patients with sHLA-G above median levels (≥ 36.8 U/ml, sHLA-G_high_) had a significantly shorter survival time than those with sHLA-G_low_ (< 36.8 U/ml, *p* < 0.001), and sHLA-G could be an independent prognostic factor for CRC patients. With stratification of clinical parameters in survival by sHLA-G_low_ and sHLA-G_high_, sHLA-G exhibited a significant predictive value for CRC patients of the female (*p* = 0.036), the elder (*p* = 0.009), advanced tumor burden (T_3 + 4_, *p* = 0.038), regional lymph node status (N_0_, *p* = 0.041), both metastasis status (M_0_, *p* = 0.014) and (M1, p=0.018), and clinical stage (I + II, *p* = 0.018), respectively. Summary, our data demonstrated for the first time that sHLA-G levels is an independent prognosis factor and improves the prognostic stratification offered by traditional prognosticators in CRC patients.

## INTRODUCTION

In China, CRC incidence and mortality have been increasing during the last decade, resulting in an estimated 376,300 new cases and 191,000 deaths in 2015 [1]. The immune system has proven to play critical roles in tumorigenesis. Various strategies such as induction of regulatory cells, alteration of antigen presentation and production of immune suppressive mediators, have been developed by tumor cells to have a successful immune evasion [2]. For the importance of the host immune system involved in tumor progression, previous literatures have demonstrated the impact of immune-classification (termed Immunoscore), and its prognostic value has been demonstrated superior to the classical TNM classification for CRC [3–5].

HLA-G, a potent immune suppressive mediator firstly observed in cytotrophoblasts, has been observed in various malignancies and strongly associated with tumor immune escape, metastasis and patient survival [6]. HLA-G can be expressed as seven different isoforms, including four membrane bound (HLA-G1 to -G4) and three soluble (HLA-G5 to -G7) molecules. Previous studies revealed that both membrane-bound and sHLA-G isoforms could render multiple immune suppressive effects during the progression of malignancies, with involved mechanisms by inhibiting immune cell function, inducing apoptosis and the generation of regulatory cells through receptor binding and/or trogocytosis, and impairing chemotaxis of different immune effector cells [7, 8].

HLA-G expression was observed in sources such as on the tumor cell, secreted, or in tumor-derived exosomes [9, 10]. A high frequency of tumor cell HLA-G expression and/or increased sHLA-G levels has been found in various body fluids in a variety of cancers [11]. An increased lesion HLA-G expression or peripheral sHLA-G levels were associated with clinical parameters such as advanced disease stage, tumor metastasis and/or worse prognosis in tumor patients [12–14]. In addition to the tumor lesion HLA-G expression was intensively investigated; however, the value of peripheral sHLA-G in prognosis is very limited but now emerging [8, 9].

In this context, previous studies revealed that sHLA-G could be a good diagnostic factor to distinguish benign colorectal related disease from CRC [15]. In gastric cancer (GC), our study indicated that plasma sHLA-G level was a potential biomarker for GC diagnosis [16]. Moreover, circulation sHLA-G levels is an independent risk factor for patients with non small cell lung cancer (NSCLC) was reported in previous studies [17–19].

In this study, plasma sHLA-G levels in 178 CRC patients were tested, and its correlation to clinical parameters and prognosis of the CRC patients was investigated.

## RESULTS

### Plasma sHLA-G levels in CRC patients

**Figure 1:**
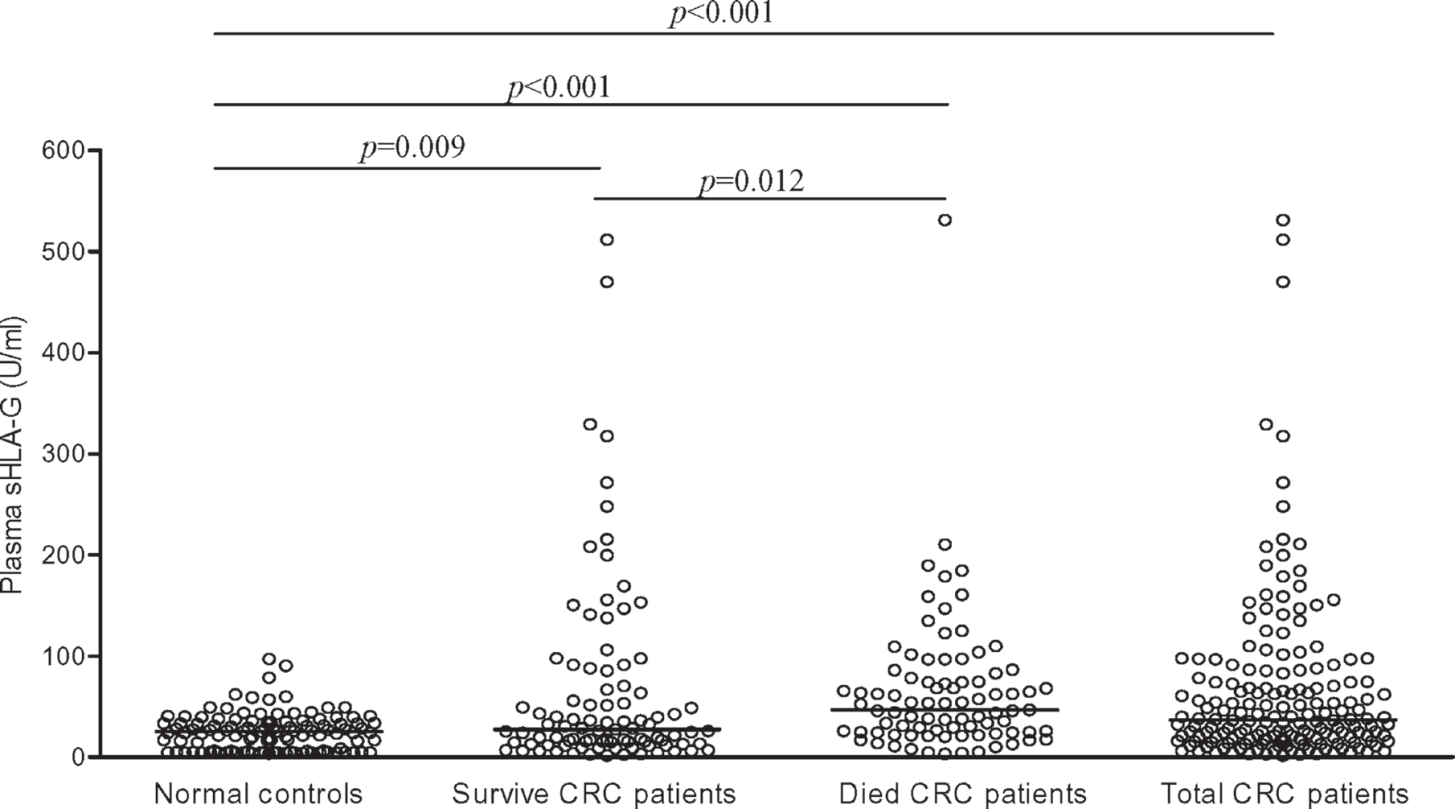
sHLA-G levels in healthy controls and CRC patients Bars represent the median values.

In CRC patients, sHLA-G (median: 36.8 U/ml; range: 1.6 – 531.0) were significantly increased compared with normal controls (median: 25.4 U/ml; range: 3.6–97.1; *p* = 0.009). Furthermore, significantly higher sHLA-G were observed in the dead (46.8 U/ml, range: 3.3–531.0) than that in the alive CRC patients (27.4 U/ml, range: 1.6–511.4; *p* = 0.012; Figure 1). However, sHLA-G were not associated with the tumor type, patient gender, age, TNM status and disease stage (Table 1).

**Table 1:** Association of sHLA-G expression with clinicopathological parameters in colorectal cancer patients

Variables	No. of cases	sHLA-G median (range, U/ml)	p*
Colorectal cancer patients	178	36.8 (1.6–531.0)	
Tumor type			
colon cancer	59	45.8 (3.3–531.0)	0.125
rectal cancer	119	35.7 (1.6–215.4)	
Survival status			
dead	85	46.8 (3.3–531.0)	0.012
alive	93	27.4 (1.6–511.4)	
Gender			
male	100	33.0 (2.8–317.5)	
female	78	43.3 (1.6–531.0)	0.122
Age			
≤ median (65 years)	95	33.7 (2.8–511.4)	
> median	83	45.8 (1.6–531.0)	0.109
T category			
T_1 + 2_	27	32.5 (1.6–511.4)	
T_3 + 4_	142	37.6 (3.2–531.0)	0.243
N category			
N_0_	79	34.3 (1.6–511.4)	0.263
N_1 + 2_	90	40.1 (3.2–531.0)	
M category			
M_0_	163	36.6 (1.6–531.0)	0.324
M_1_	6	17.8 (5.2 −134.9)	
Disease stage			
I	24	33.4 (1.6–511.4)	0.582
II	54	36.2 (3.3 −469.9)	
III	85	40.1 (3.2–531.0)	
IV	6	17.8 (5.2 −134.9)	

*Comparison of sHLA-G expression status between or among each variable using the Mann-Whitney *U* test.

### Plasma sHLA-G related to survival in CRC patients

To investigate the relationship between plasma sHLA-G and the survival of the CRC patients, sHLA-G was stratified to two groups as below (sHLA-G_low_) or above (sHLA-G_high_) the median of 36.8 U/ml. The significance of other factors for the prognosis such as patient gender, age, extent of primary tumor (T), regional lymphnode status (N), distant metastases (M), and disease stage was also analyzed.

**Figure 2:**
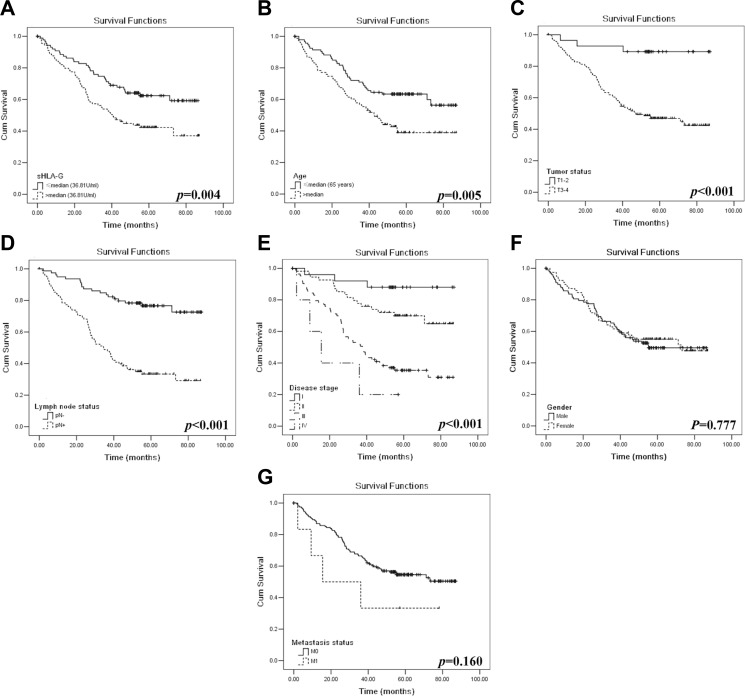
Kaplan–Meier survival analysis for CRC patients Comparison of the overall survival between the (**A**) patients with sHLA-G_high_ (*n* = 89) and sHLA-G_low_ (*n* = 89); (**B**) patients with age above (*n* = 83) and below (*n* = 95) the median of 65 years; (**C**) T_1 + 2_ (*n* = 27) and T_3+4_ (*n* = 142); (**D**) N_0_ (*n* = 79) and N_1+2_ (*n* = 90); (**E**) disease stages I (*n* = 25), II (*n* = 54), III (*n* = 85) and IV (*n* = 6); (**F**) male (*n* = 100) and female (*n* = 78) patients, and (**G**) M_0_ (*n* = 163) and M_1_ (*n* = 6) of CRC patients.

CRC patients with sHLA-G_high_ (*n* = 89) had a significantly worse prognosis than patients with sHLA-G_low_ (*n* = 89; *p* = 0.004). The median survival time for the sHLA-G_low_ patients was 62.9 months (95% CI: 56.3–69.5), and for the sHLA-G_high_ patients was 49.2 months (95% CI: 42.2–56.1; Figure 2A).

Other factors including patient age, T and N status, and disease stage was found significantly associated with survival. Patients with the age below (*n* = 95) had a notably longer survival than those with the age above the median of 65 years [*n* = 83; median: 62.4 months (95% CI: 56.0–68.8) *vs* 48.8 months (95% CI: 41.61 – 156.1), *p* = 0.005; Figure 2B]. Primary tumor status T_1+2_ (*n* = 27) had a notably longer survival than those with T_3+4_ [*n* = 142; median: 80.0 months (95% CI: 72.2–87.8) *vs* 53.0 months (95% CI: 47.6–58.5); *p* < 0.001; Figure 2C]. Patients with N_0_ (*n* = 79) had a longer survival than those with N_1 + 2_ [*n* = 90; median: 72.4 months (95% CI: 66.5–78.3) *vs* 44.0 months (95% CI: 37.4–50.7; *p <* 0.001; Figure 2D]. Moreover, Patients with advanced disease stage had a remarkably shorter survival time (*p* < 0.001), with the survival time for stage I [*n* = 24; median: 81.8 months; (95% CI: 74.5–89.1)], II [*n* = 54; median: 68.0 months; (95% CI: 60.4–75.7)], III [*n* = 85; median: 44.8 months; (95% CI: 38.0–51.6)], and stage IV [*n* = 6; median: 24.0 months; (95% CI: 6.5–41.5)], respectively (Figure 2E). However, no statistical difference was observed between the male (*n* = 100) and female patients (*n* = 78) (median: 55.3 months *vs* 56.9 months; *p* = 0.777; Figure 2F), and similar data was found for tumor metastasis status, though the survival is much longer in patients with M_0_ (*n* = 163) than those with M_1_ (*n* = 6; median: 58.2 months *vs* 36.6 months; *p* = 0.160; Figure 2G).

To evaluate whether sHLA-G is a prognostic factor for CRC patients, Cox's proportional hazards model analysis was performed. In addition to sHLA-G, clinicopathological parameters including patient age, gender, TNM status and disease stage was included. Univariate analysis showed that variates such as sHLA-G (*p* = 0.005, HR = 1.870), patient age (*p* = 0.006, HR = 1.830), T status (*p* < 0.001, HR=3.525), N status (*p <* 0.001, HR=4.021) and disease stage (*p* < 0.001, HR = 3.887), all were significantly to a poor prognosis. Moreover, multivariate analysis revealed that, besides the patient age and primary tumor status, sHLA-G was an independent prognostic factor (*p* = 0.047, HR=1.622). These results indicated that the sHLA-G was an independent prognostic factor for CRC patients (Table 2).

**Table 2:** Cox proportional hazards model analysis of variables affecting survival in colorectal cancer patients

Variables	Categories	Univariate Analysis		Multivariate Analysis	
		Overall survival		Overall survival	
		HR (95% CI)	*P*-value	HR (95% CI)	*P*-value
Gender	Male (*vs* female)	0.940 (0.612–1.443)	0.776		
Age (years)	> 65 (*vs* ≤ 65)	1.830 (1.189–2.816)	0.006	1.909 (1.202–3.033)	0.006
T category	T_3 + 4_ (*vs* T_1 + 2_)	3.525 (1.898–6.547)	< 0.001	2.044 (1.062–3.934)	0.032
N category	N_1 + 2_ (*vs* N_0_)	4.021 (2.391–6.764)	< 0.001	1.759 (0.409–7.573)	0.448
M category	M_1_ (*vs* M_0_)	2.029 (0.741–5.554)	0.168	1.701 (0.541–5.349)	0.363
Disease stage	III/IV (*vs* I/II)	3.887 (2.311–6.538)	< 0.001	1.833 (0.436–7.709)	0.408
sHLA-G (U/ml)	> 36.8 (*vs* ≤ 36.8)	1.870 (1.207–2.897)	0.005	1.622 (1.006–2.615)	0.047

Abbreviations: HR = hazard ratio; 95% CI = 95% confidence interval; TNM, lymph-node-metastasis and disease stage according to the TNM classification for colorectal cancer (UICC).

For multiple comparisons are done including T, N, and disease stage, Bonferroni correction was performed. Because only 3 patients were T_1_ and 5 patients were T_4_, Bonferroni correction was not performed. Among the N status, data showed that survival for patients with N_1_ and N_2_ was worse than patients with N_0_ (all *p*_c_ < 0.001), while no significance was observed between the N_1_ and N_2_ (*p*_c_ = 0.717). Survival for the disease stages, patients with III and IV were worse than those with I (*p*_c_ = 0.004 and *p*_c_ < 0.001) and II (*p*_c_ < 0.001 and *p*_c_ = 0.088), respectively. However, survival between patient with I and II (*p*_c_ = 0.168), III and IV (*p* = 0.746) were not significant (Table 3).

**Table 3:** Log-rank Mantel-Cox analysis of multi-variables affecting survival in colorectal cancer patients

Variables	No. Total	No. Events	Survival time Mean (95% CI)	*P*-value*	HR (95% CI)	*P*-value*
**N category**						
Whole cohort	169	78	57.5 (52.6–62.5)	*p*_c_ < 0.001	2.110 (1.615–2.755)	*p*_c_ < 0.001
N_0_	79	19	72.4 (66.5–78.3)			
N_1_	49	30	46.4 (37.8–55.0)			
N_2_	41	29	39.7 (30.0–49.4)			
N_1_ *vs*. N_0_				*p*_c_ < 0.001	3.546 (1.989–6.321)	*p*_c_ < 0.001
N_2_ *vs*. N_0_				*p*_c_ < 0.001	2.164 (1.615–2.899)	*p*_c_ < 0.001
N_2_ *vs*. N_1_				*p*_c_ = 0.717	1.358 (0.814–2.265)	*p*_c_ = 0.723
**Disease stage**						
Whole cohort	169	78	57.5 (52.6–62.5)	*p*_c_ < 0.001	2.490 (1.789–3.467)	*p*_c_ < 0.001
I	24	2	81.8 (74.5–89.1)			
II	54	17	68.0 (60.4–75.7)			
III	85	55	44.8 (38.0–51.6)			
IV	6	4	24.0 (6.50–41.5)			
II *vs*. I				*p*_c_ = 0.168	4.068 (0.939–17.63)	*p*_c_ = 0.244
III *vs*. I				*p*_c_ = 0.004	3.487 (1.721–7.067)	*p*_c_ = 0.004
IV *vs*. I				*p*_c_ < 0.001	2.299 (1.294–4.083)	*p*_c_ = 0.020
III *vs*. II				*p*_c_ < 0.001	2.912 (1.685–5.033)	*p*_c_ < 0.001
IV *vs*. II				*p*_c_ = 0.088	1.823 (1.056–3.149)	*p*_c_ = 0.124
IV *vs*. III				*P* = 0.746	1.182 (0.428–3.267)	*P* = 0.747

Abbreviations: HR = hazard ratio; 95% CI = 95% confidence interval; Disease stage according to the TNM classification for colorectal cancer (UICC). *If a significant difference was found, the Bonferroni correction was performed (*p*_c_).

### sHLA-G levels on the prognostic stratification of clinical parameters in CRC patients

Then, we analyzed the prognostic significance of sHLA-G with stratification of clinical parameters in CRC patients. Briefly, patient gender were stratified to male and female, age stratified to below and above the median age (65 years), categories T stratified to T_1 + 2_ and T_3 + 4_, N stratified to N_0_ and N_1 + 2_, M stratified to M_0_ and M_1_, and clinical disease stage was stratified to I + II and III + IV, respectively.

**Figure 3:**
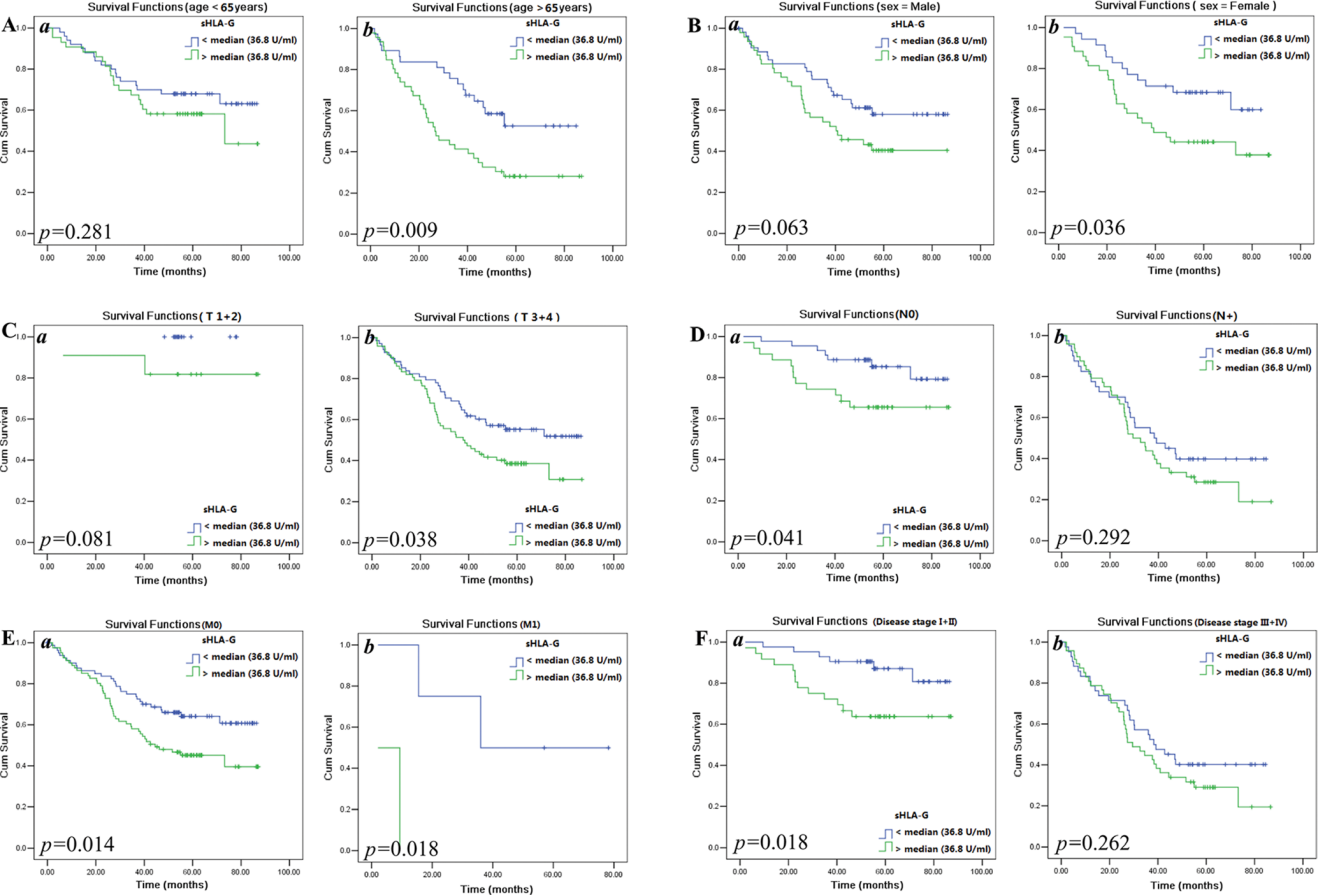
Kaplan-Meier survival analysis of stratified clinical parameters in survival by sHLA-G (sHLA-G_low_ or sHLA-G_high_) in CRC patients, respectively Stratified clinical parameters (**A**) patients with age below or above the median of 65 years; (**B**) male or female patients; (**C**) T_1+2_ or T_3+4_; (**D**) N_0_ and N_1+2_; (**E**) M_0_ or M_1_; and (**F**) disease stage I + II or III + IV.

Data showed that sHLA-G levels could significantly affects the CRC patient survival when clinical parameters were stratified. The detail results were shown in Table 4. The elder patients have poorer survival with sHLA-G_high_ than those sHLA-G_low_ (*p* = 0.009; Figure 3Ab). Similarly, female patients with sHLA-G high have a significantly shoter survival than those with sHLA-G_low_ (*p* = 0.036; Figure 3Bb). Moreover, patients with sHLA-G_high_ exhibited a significant predictive power for CRC patients with T_3+4_ (*p* = 0.038; Figure 3Cb), N_0_ (*p* = 0.041; Figure 3Da), both M_0_ (*p* = 0.014; Figure 3Ea)) and M_1_ (*p* = 0.018; Figure 3Eb), and clinical stage (I + II, *p* = 0.018; Figure 3Fa), respectively.

**Table 4:** Log-rank Mantel-Cox analysis of stratified variables in survival by plasma sHLA-G levels in CRC patients

Variables	Stratified variables	Whole cohort				sHLA-G <36.8 U/ml			sHLA-G >36.8 U/ml			
		No. Total	No. Events	Survival time Mean (95% CI)	*p*	No. Total	No. Events	Survival time Mean (95% CI)	No. Total	No. Events	Survival time Mean (95% CI)	*p*
Gender	Male	100	48	55.3 (48.7-61.9)	0.777	54	21	61.0 (52.2-69.8)	46	27	48.9 (39.3-58.4)	0.063
	Female	78	37	56.9 (49.6-64.2)		35	12	63.8 (54.4-73.1)	43	25	49.7 (39.7-59.7)	0.036
Age	≤65 ys	95	36	62.4 (56.0-68.8)	0.005	52	17	65.1 (56.6-73.6)	43	19	58.4 (48.7-68.1)	0.281
	>65 ys	83	49	48.8 (41.6-56.1)		37	16	58.6 (48.4-68.7)	46	33	40.2 (30.9-49.4)	0.009
Tumor status	T_1+2_	27	2	82.4 (75.9-88.9)	<0.001	16	0	/	11	2	/	0.081
	T_3+4_	142	76	52.7 (47.3-58.2)		70	31	58.1 (50.4-65.8)	72	45	47.2 (39.8-54.6)	0.038
Nodal status	N_0_	79	19	72.4 (66.5-78.3)	<0.001	44	7	77.8 (71.8-83.8)	35	12	65.2 (54.8-75.6)	0.041
	N_1+2_	90	59	44.0 (37.4-50.7)		42	24	46.9 (36.8-57.0)	48	35	40.8 (32.4-49.2)	0.290
Metastasis status	M_0_	163	74	58.2 (53.2-63.2)	0.160	82	29	63.7 (56.9-70.6)	81	45	52.3 (45.2-59.3)	0.014
	M_1_	6	4	36.6 (11.6-61.5)		4	2	51.9 (25.3-78.6)	2	2	5.70 (0.00-12.8)	0.018
Disease stage	I+II	78	19	72.2 (66.2-78.2)	<0.001	42	6	78.6 (72.6-84.5)	36	13	64.3 (54.1-74.6)	0.018
	III+IV	91	59	44.5 (37.9-51.1)		44	25	47.5 (37.7-57.3)	47	34	41.0 (32.4-49.5)	0.262

Abbreviations: 95% CI = 95% confidence interval; TNM, lymph-node-metastasis and stage according to the TNM classification.

## DISCUSSION

The aberrant HLA-G expression as a clinical biomarker for diagnosis or prognosis has been intensively investigated in tumors [8]. Both membrane-bound and sHLA-G proteins have similar immune suppression functions by directly binding to specific receptors such as immunoglobulin-like transcripts-2 and -4 expressed on immune cells [20, 21]. HLA-G could also induce regulatory CD4+CD25+FoxP3+ T cells, B cells, DCs, NK cells and MDSCs, which provided these immune effectors with a long term immunomodulatory function [22–25].

Given to its immune suppressive property, peripheral sHLA-G could impair host antitumor immune response either locally at the tumor site or systemically via the circulation. Previous studies revealed that plasma sHLA-G are significantly increased in patients with cancers such as lung cancer, breast and ovarian carcinoma as well as in patients with leukemia [26–28]. Beyond its suppressive immune functions, sHLA-G was considered as a diagnostic tool to distinguish between malignant and benign tumors or health controls, and as a prognostic factor in prediction of the disease outcome [16–18, 29].

In this scenario, sHLA-G was significantly increased in CRC patients, and sHLA-G is a powerful item to distinguish CRC from benign colorectal diseases, and the combination of sHLA-G and carcinoembryonic antigen showed a higher detection capacity than individual markers alone [15]. sHLA-G was also showed as a better diagnostic factor than carbohydrate antigen 125 in cervical and gastric cancer patients [16, 30]. In another study, sHLA-G was found exclusively elevated in NSCLC and sHLA-G could be a potent predictor for prognosis, that patients with sHLA-G less than 40 ng/ml have a significantly better survival [18]. In NSCLC patients, our study showed that increased sHLA-G was associated with the advanced disease stage and poor survival [19]. A recent study by Ben Amor et al. [17]. also showed that NSCLC patients with sHLA-G above median level had a significantly shorter survival time and sHLA-G was an independent risk factor for NSCLC patients.

In this study, sHLA-G was significantly increased in CRC patients than that in normal controls, and much higher sHLA-G was observed in died than that in alive CRC patients. More importantly, CRC patients with sHLA-G_high_ had a statistically significant shorter survival time than those with sHLA-G_low_. In this context, other studies showed that with sHLA-G above 32 U/ml, 40 U/ml and 50 U/ml were associated with a poor prognosis in NSCLC patients [17–19].

To be noted, clinical outcome significantly varies among patients within the same disease stage; however, the ‘Immunoscore’ components such as CD3+, CD8+ and CD45RO+ T cell infiltration incorporating into traditional classification could improve classical TNM prognostic power [31]. Considering HLA-G is a powerful immune inhibitory antigen [8], whether sHLA-G could improve the performance of traditional predictors in CRC was evaluated in this study. Our study showed sHLA-G could significantly affects the CRC survival when traditional clinical parameters and prognosis predictors were stratified. In the group of sHLA-G_high_, we found that the female, the elder, and patients with T_3+4_, N_0_, M_0_ and M_1_, and disease stage I+II, have dramatically poor survival than those with sHLA-G_low_.

Taken together, our study revealed that, besides sHLA-G could be an independent prognosis factor, the combination of sHLA-G with other traditional risk factors could improve their prognostic values for the particular subpopulations of CRC patients. Given its immune inhibitory property and prognostic value, sHLA-G in patients with CRC might be a new component for the ‘Immunescore’, contributing an additional significance to the classical cancer TNM classification system.

## MATERIALS AND METHODS

### Patients and samples

From April 2007 to May 2013, 178 plasma samples before surgery were consecutively collected from Chinese Han CRC patients (100 males and 78 females, aged from 28 years to 86 years), who were diagnosed and treated at Taizhou Hospital of Zhejiang Province, Wenzhou Medical University. Only patients with histopathologically confirmed of CRC were included in this study. None of the patients received radiotherapy, chemotherapy, or other medical interventions before blood sampling. Patient data including age, gender, date of initial diagnosis, TNM and disease stage were documented. The disease stage was determined according to the 7^th^ TNM staging system by International Union for Cancer Control (UICC) and the American Joint Committee for Cancer (AJCC) [32].

Of 178 cases, the TNM status of 169 cases was available, where 24, 54, 85 and 6 patients with disease stage I, II, III and IV, respectively. Among these cases, 176 patients were available for the follow-up study till the last follow-up date at 25^th^, September 2014. Overall patient survival was defined as the time from the date of surgery to the date of last follow-up (censored) or patient death (event). The median follow-up for all patients was 47 months (range, 2–91), and during the entire period, there were 85 cancer-related deaths including 2, 17, 55 and 4 patients with stage I, II, III and IV, respectively.

Plasma samples from 113 sex- and age-matched (69 males and 44 females, aged from 22 years to 82 years), unrelated healthy Chinese Han individuals were served as normal controls. Plasma samples of patients and controls were prepared from the peripheral blood by centrifugation at 1500 *g* for 10 minutes, and stored at −80°C until use. Written informed consent was obtained from each individual, and this study was performed following an Institutional Ethics Review Board approved protocol to investigate molecular markers relevant to CRC pathogenesis.

### sHLA-G enzymed-linked immunosorbent assay (ELISA)

sHLA-G levels in plasma from 178 CRC patients and 113 unrelated healthy blood donors were determined. sHLA-G concentrations were determined with the sHLA-G specific ELISA kit (sHLA-G kit; Exbio, Prague, Czech Republic), which measures sHLA-G1 and HLA-G5. The optical densities were measured at 450 nm (Spectra Max 250, Molecular Devices, Sunnyvale, CA). The final concentration was determined by optical density according to the standard curves. The detection limits were 1 U/ml. Details of the performance were according to the manufacture's instruction.

### Statistical analysis

Statistical analysis was performed with SPSS 13.0 software (SPSS, Inc., Chicago, IL). Difference of sHLA-G between groups was analyzed with Mann-Whitney *U-test*. Overall patient survival was evaluated from the date of surgery to the event of interest or censored on the last follow-up. Survival probabilities were calculated using the Kaplan-Meier method. Differences between survival curves were analyzed by the log-rank test. Univariate and multivariate Cox regression analysis was used to define clinicopathological variables as independent predictors for overall survival. *p <* 0.05 was considered significant.
